# Supplementary material for: Complexome profiling of the Chlamydomonas psb28 mutant reveals TEF5 as an early PSII assembly factor
Source: Plant Cell. 2025 Mar 17;37(6):koaf055. doi: 10.1093/plcell/koaf055 (PMC12164590; doi:10.1093/plcell/koaf055)
Supplement: koaf055_Supplementary_Data [file koaf055_supplementary_data.zip › psb28_interactive.zip!/plots/intensity/qualityPlots/peptides/COX3_Cre04.g221700.t1.2.html]

### Cre04.g221700.t1.2

Trivial name: COX3  
SeqCoverage [median]: 10.70  
Mass: 41.42 kDa  
  
 

| Sequence | MissedCleavage | Mass [Da] | wtIntensity | muIntensity | Score |
| --- | --- | --- | --- | --- | --- |
| ALVMSAVLAASYYSANVAMVAK | 0 | 2229.15 | 1260.28 | 0.00 | 138.07 |
| EFYMEHIGK | 0 | 1152.53 | 2514.09 | 5394.45 | 56.81 |
| EFYMEHIGKR | 1 | 1308.63 | 15206.68 | 15166.56 | 73.23 |
| THNAGAALK | 0 | 881.47 | 24217.02 | 24086.53 | 178.30 |
|  |  |  | 43198.1 | 44647.5 |  |
